# Effect of dehydration on the inactivation of *Listeria monocytogenes* and *Salmonella enterica* on enoki and wood ear mushrooms

**DOI:** 10.3389/fmicb.2023.1257053

**Published:** 2023-10-31

**Authors:** Joelle K. Salazar, Megan L. Fay, Bashayer A. Khouja, Nirali J. Chavda, Gayatri R. Patil, David T. Ingram

**Affiliations:** ^1^Division of Food Processing Science and Technology, U. S. Food and Drug Administration, Bedford Park, IL, United States; ^2^Department of Food Science and Nutrition, Illinois Institute of Technology, Bedford Park, IL, United States; ^3^Division of Produce Safety, U. S. Food and Drug Administration, College Park, MD, United States

**Keywords:** fungi, dehydration, heat treatment, inactivation kinetics, *Listeria*, *Salmonella*

## Abstract

Foodborne illness outbreaks in the U.S. associated with consumption of both fresh and dried specialty mushrooms have recently occurred. Dried wood ear mushrooms were implicated in a salmonellosis outbreak in 2020, while fresh enoki mushrooms were associated with two listeriosis outbreaks in 2020 and 2023. These specialty mushrooms are commercially available in both their fresh and dried states. Due to the short shelf life of mushrooms, dehydration is a common method used in both industry and by consumers to extend the shelf life and preserve quality. Therefore, the aim of this study was to evaluate the use of dehydration on the inactivation kinetics of both *Listeria monocytogenes* and *Salmonella enterica* on enoki and wood ear mushrooms. Fresh mushrooms were inoculated with four strain cocktails of either *L. monocytogenes* or *S. enterica* and dried at ambient conditions for 10 min. Following drying of the inoculum, mushrooms were placed into food dehydrators preheated to 70, 80, or 90°C and treated for up to 24 h. At treatment intervals, mushrooms were removed from the dehydrators for pathogen enumeration. Inactivation kinetics for both pathogens were modeled using the Weibull, log-linear with tail, and log-linear with shoulder models. Pathogen reductions of >4 log CFU/g were achieved on both enoki and wood ear mushrooms during dehydration at 90°C after only 2–4 h. At 70 and 80°C, log reductions of >4 log CFU/g were observed on wood ear mushrooms after 4–8 h. On enoki mushrooms, a tailing effect was observed with residual populations (>2 log CFU/g) of *L. monocytogenes* and *S. enterica* remaining even after 24 h of treatment at both 70 and 80°C. This study emphasizes the need for an individualized dehydration strategy for each mushroom type to ensure the effectiveness of dehydration as a process to reduce pathogen populations. Results of this study will aid in informing proper time and temperature combinations for dehydration of specialty mushrooms to ensure product safety.

## 1. Introduction

Foodborne outbreaks in the U.S. associated with specialty mushrooms have occurred in recent years (U.S. Centers for Disease Control and Prevention, 2020a,b; U.S. Food and Drug Administration, 2020a,b; U.S. Centers for Disease Control and Prevention, 2023). In 2020, a salmonellosis outbreak linked to dried wood ear mushrooms occurred causing 55 illnesses across 12 states which resulted in six hospitalizations (U.S. Centers for Disease Control and Prevention, 2020a; U.S. Food and Drug Administration, 2020a). The implicated dried wood ear mushrooms were sold in bulk to restaurants and were not available directly to consumers for purchase. A majority of the ill individuals who were interviewed reported consuming ramen soup at restaurants and the menus of the restaurants indicated that wood ear mushrooms, or kikurage, were ingredients in the ramen. Two other outbreaks linked to enoki mushrooms occurred in 2020 and 2023 (U.S. Centers for Disease Control and Prevention, 2020b; U.S. Food and Drug Administration, 2020b; U.S. Centers for Disease Control and Prevention, 2023). First, in 2020, a listeriosis outbreak caused 36 illnesses across 17 states, resulting in 31 hospitalizations and four deaths (U.S. Centers for Disease Control and Prevention, 2020b; U.S. Food and Drug Administration, 2020b). Secondly, in 2023, another smaller listeriosis outbreak occurred resulting in five illnesses across four states (U.S. Centers for Disease Control and Prevention, 2023). All of the ill individuals were hospitalized. The implicated enoki mushrooms in both outbreaks were imported from Asian countries and sold directly to consumers at retail establishments. Testing by the U.S. Food and Drug Administration determined that 43% of the enoki mushrooms imported from the Republic of Korea were contaminated with *L. monocytogenes* (U.S. Food and Drug Administration, 2022).

Specialty mushrooms are readily commercially available in their fresh or dried states. The implicated wood ear mushrooms in the salmonellosis outbreak were dried and sold in bulk to restaurants. Since mushrooms have relatively short shelf lives (approximately 1–3 days at ambient and up to 14 days at refrigeration temperatures under certain storage conditions) (Fernandes et al., 2012), various methods are used to prolong the shelf life and preserve quality. Drying is one common method used to reduce the water activity (a_w_) to <0.70 and thus prevent the proliferation of microorganisms, including bacteria. Drying mushrooms is also convenient for transportation and storage as the product requires a smaller footprint and its weight is greatly reduced. Nonetheless, drying methods relying on heated air are often conducted at low temperatures (<80°C) for short periods of time in order to retain the organoleptic properties of the product (Bourdoux et al., 2016). In an industry setting, vegetables and mushrooms are commonly dried using a tunnel of hot air, where the internal product temperature often remains below 45°C due to water evaporation (Fernandes et al., 2012). Therefore, as a standalone method, drying under these low temperature and short time conditions may not be considered a preventive control to substantially reduce or eliminate any populations of bacterial pathogens potentially present on the fresh mushrooms.

Even though the enoki mushrooms involved in both listeriosis outbreaks were sold to consumers in their fresh state, enoki mushrooms, as with other types of specialty mushrooms, are also commercially available in their dried state. Consumers may also choose to dry fresh mushrooms in their homes using household food dehydrators. Consumers looking for instructions on how to dehydrate mushrooms may find information online at educational extension websites (Utah State University Extension, 1994; Montana State University Extension, 2017; University of Minnesota Extension, 2020; Colorado State University Extension, 2023) suggesting a “low and slow” approach focusing on the integrity of the dried product. These websites suggest drying mushrooms in household food dehydrators at low temperatures (≤60°C) for short time periods (3.5–10 h). While these temperature and time combinations would be sufficient to dry mushrooms to an a_w_ < 0.70, where no pathogen growth would occur, minimal reductions in pathogen populations due to drying would be expected.

Information on the survival or inactivation of foodborne bacterial pathogens on mushrooms during drying at moderate to high temperatures are lacking. One published study evaluated the survival of *L. monocytogenes* and *Salmonella enterica* serovar Typhimurium on sliced portobello mushrooms during hot-air drying in a household food dehydrator (Kragh et al., 2022). The study determined that dehydration of the mushrooms at 55°C (air temperature 46°C and product internal temperature 40–43°C) resulted in log reductions of only 2.6 and 2.5 for *L. monocytogenes* and *S.* Typhimurium, respectively, after 8 h. Similar or lower log reductions of *S. enterica* were observed by other studies on sliced apples (2.7–4.0 log) and carrots (1.6–1.7 log) during dehydration at 60°C for 6 h in household food dehydrators (Dipersio et al., 2003, 2007). Pathogen inactivation appears to be dependent on not only the dehydration temperature and time, but also on the intrinsic properties of the produce item, including initial moisture content.

The objective of this study was to evaluate the inactivation kinetics of both *L. monocytogenes* and *S. enterica* on fresh enoki and wood ear mushrooms during dehydration at different temperatures. Since both of these specialty mushroom types are available to consumers in their dehydrated states, understanding how and to what extent the dehydration process effects pathogen inactivation on these products is needed. Results of this study will aid in informing proper time and temperature combinations for specialty mushroom dehydration to ensure product safety.

## 2. Materials and methods

### 2.1. Mushroom preparation

Fresh raw enoki and wood ear mushrooms were acquired from local retail grocers in Illinois and stored at 5°C for up to 24 h prior to use. For the enoki mushrooms, the bottom 3 cm containing growth substrate was removed. Both mushroom types were chopped: enoki pieces measured 2.5 cm long and wood ear pieces measured 2.5 cm × 2.5 cm. Chopped mushrooms (10 g) were weighed into 6-cm foil pans.

### 2.2. Strains and culture conditions

A four-strain cocktail of either *L. monocytogenes* or *S. enterica* was used. The *L. monocytogenes* strains included LS806 (isolated from hummus), LS3132 (isolated from avocado), LS0352 (isolated from cream cheese), and ScottA [clinical isolate (Briers et al., 2011)]. The *S. enterica* strains included Enteritidis PT30 (ATCC BAA-1045, almonds isolate), Agona [447967, roasted oats cereal isolate (U.S. Centers for Disease Control and Prevention, 1998)], Alachua [CFSAN107331, peach leaf isolate (U.S. Food and Drug Administration, 2020c)], and Poona 8785 (CFSAN038692, cucumber isolate). All strains were rifampicin resistant (100 μg/mL) and selected due to their strong desiccation resistance profiles. Each strain was cultured individually in Tryptic Soy Broth (TSB; Becton, Dickinson and Co., Sparks, MD) for 16–18 h at 37°C. The cultures were then washed twice with Butterfields’s Phosphate Buffer (BPB, pH 7.2), resuspended in BPB, and combined in equal volumes to create a four-strain cocktail of either *L. monocytogenes* or *S. enterica* (9 log CFU/mL). The cocktails were serially diluted to 8 log CFU/mL and used immediately for mushroom inoculation. To verify the initial population levels, both cocktails were serially diluted in BPB and plated onto Brain Heart Infusion Agar (BHIA; Becton, Dickinson and Co.). Agar plates were incubated at 37°C for 24–48 h.

### 2.3. Mushroom inoculation and dehydration

Mushrooms (10 g per sample) were placed into aluminum foil trays (60 mm) and spot inoculated with the diluted *L. monocytogenes* or *S. enterica* cocktail (10 spots of 10 μL each for each sample) at approximately 6.5 log CFU/g. Inoculated mushrooms were dried at ambient for 10 min. Five household food dehydrators (Septree Food Dehydrator, model DCS-04A) were pre-heated for 10 min at 70, 80, or 90°C. Triplicate mushroom samples were placed onto the middle shelf in the pre-heated dehydrators. The circulating air temperature at the center of the middle shelf as well as the internal temperature of the mushrooms during dehydration was continuously monitored using probe thermometers coupled to a data logger (Fluke 52 II, Fluke Corporation, Everett, WA). To avoid temperature fluctuations due to opening the dehydrator doors, only one sampling timepoint was conducted per dehydrator at one time and a different dehydrator was used for each trial. Sampling timepoints included 0, 0.5, 1, 1.5, 2, 4, 6, 8, and 24 h. Triplicate samples were assessed at each timepoint and three independent trials were conducted (n = 9).

### 2.4. Enumeration of *Listeria monocytogenes* and *Salmonella enterica*

*L. monocytogenes and S. enterica* were enumerated after each dehydration timepoint. Mushrooms (initial weight of 10 g) were homogenized 1:10 with Buffered *Listeria* Enrichment Broth (BLEB; Becton, Dickinson and Co.) or BPB, for *L. monocytogenes and S. enterica* respectively, for 1 min in at 185 rpm a stomacher (Stomacher 400 Circulator, Seward, United Kingdom). Homogenates were serially diluted and plated onto BHIA supplemented with 100 μg/mL of rifampicin (BHIA^rif^) for enumeration. All agar plates were incubated at 37°C for 24–48 h. Data were expressed as log CFU/g per initial 10-g sample.

### 2.5. Measurement of moisture content

The moisture content of the freshly chopped enoki and wood ear mushrooms (10 g) and the mushrooms after each dehydration sampling timepoint were assessed. To determine the moisture content of the freshly chopped mushrooms, the samples were dried in an oven at 100°C for 24 h and the remaining solid weight was measured. After each dehydration timepoint, the samples were also measured to determine the remining solid weight. Moisture content results were reported on a wet basis. Triplicate samples were assessed at each timepoint and three independent trials were conducted (*n* = 9).

### 2.6. Primary modeling

The populations (log CFU/g) of *L. monocytogenes* and *S. enterica* on both enoki and wood ear mushrooms (10 g initial weight) during dehydration at 70, 80, and 90°C were fitted to the Weibull model (Mafart et al., 2002). Populations during dehydration at 70 and 80°C were also fitted to the log-linear with tail model, while populations during dehydration at 90°C were also fitted to the log-linear with shoulder model (Geeraerd et al., 2000). All modeling was conducted using the GInaFiT v 1.6 add-in for Excel (Geeraerd et al., 2005).

The Weibull model is displayed in Eq. 1 (Mafart et al., 2002). This model assumes that the resistance to stress of a microbial population follows the Weibull distribution.


(1) $$\log_{10}\;N\left(t\right)=\log_{10}\;N_{0}-{\left(t/\delta \right)}^{\rho }$$

where *N (t)* is the population at time *t*, *N*_0_ is the initial population, *ρ* is the shape parameter (*ρ* = 1 describes log-linear, *ρ* < 1 describes concave, and *ρ* > 1 describes convex), and δ is the time for the first decimal reduction in microbial population. This model is commonly used when examining microbial inactivation and has been shown to provide the best fit in low a_w_ (<0.60) food matrices at a wide range of temperatures (21 to 80°C) (Farakos et al., 2013, 2017).

The log-linear with tail and the log-linear with shoulder models are displayed in Eqs 2, 3, respectively (Geeraerd et al., 2000).


(2) $$N=\left(N_{0}-N_{res}\right)\times e^{-k_{\text{max}}\times t}+N_{res}$$

(3) $$N=N_{0}\times e^{-k_{\text{max}}\times t}\times \left(\left(e^{-k_{\text{max}}\times Sl}\right)/\left(1+\left(e^{-k_{\text{max}}\times Sl-1}\right)\times e^{-k_{\text{max}}\times t}\right)\right)$$

where *N* is the population at time *t*, *N*_0_ is the initial population, *N*_res_ is the residual or remaining population, *k*_max_ is the maximum inactivation rate, and Sl is the shoulder length (time unit).

### 2.7. Statistical analysis

The moisture contents of both mushroom types after chopping and during dehydration were statistically compared using ANOVA with Tukey’s post-hoc test (*α* = 0.05). The populations of *L. monocytogenes* and *S. enterica* on both mushroom types after inoculation and during dehydration were also statistically compared using ANOVA. For primary modeling, the goodness of fit parameters, mean square error (MSE) and coefficient of determination (*r*^2^), were reported by GInaFiT.

## 3. Results

### 3.1. Temperatures and moisture contents of the mushrooms during dehydration

After pre-heating the food dehydrators to 70, 80, or 90°C for 10 min, the circulating air temperatures measured at the center shelf in the units were 68.9, 82.5, and 90.2°C, respectively. During dehydration, the air temperatures fluctuated only minimally from 68.9–70.2, 81.3–83.0, and 89.8–92.3°C, respectively. The initial temperature of the enoki and wood ear mushrooms prior to dehydration were 20.8 and 21.0°C, respectively. During dehydration at the set temperatures of 70, 80, or 90°C, the come-up time for the internal temperatures of the wood ear and enoki mushrooms to reach 68.0, 78.5, or 87.0°C were 2.0, 3.2, and 5.0 min, respectively. At 70, 80, or 90°C, the internal temperature of the wood ear mushrooms plateaued at 69.8°C after 1.75 h, 79.5°C after 1.58 h, and 87.6°C after 1 h, respectively. For the enoki mushrooms, the internal temperature plateaued at 69.9°C after 1.5 h, 80.0°C after 0.83 h, and 90°C after 0.83 h, respectively.

**Figure 1:**
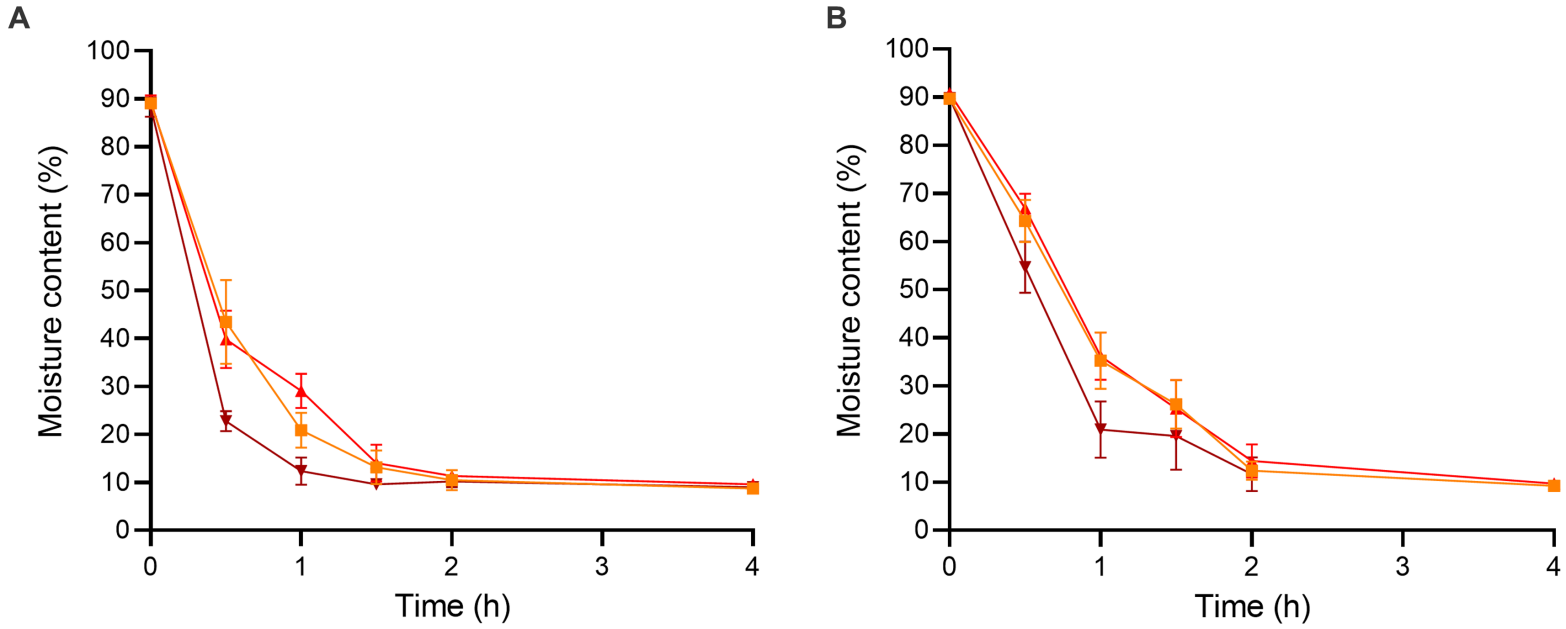
The moisture contents of the **(A)** enoki and **(B)** wood ear mushrooms during dehydration at 70 (orange square), 80 (red circle), and 90°C (brown triangle) for 4 h. Data are mean values ± standard deviation (*n* = 9).

The initial moisture contents of the enoki and wood ear mushrooms after chopping were 89.07 ± 1.07 and 89.67 ± 0.82%, respectively (Figure 1). After 0.5 h of dehydration, the moisture content of the enoki mushrooms significantly decreased at all temperatures; the moisture content at 90°C (22.76 ± 2.11%) was significantly lower than that at both 70 and 80°C. Similarly, after 1 h of dehydration, the moisture content at 90°C (12.35 ± 2.84%) was significantly lower than that at the other two temperatures. After 4 h of dehydration, the moisture content of the enoki mushrooms equilibrated across temperatures; there was no significant difference between the moisture contents at 70, 80, or 90°C (8.68 ± 0.45, 9.54 ± 0.92, and 8.97 ± 0.66%, respectively). Dehydration for longer time periods did not result in a significant decrease in the moisture content of the enoki mushrooms.

After 0.5 h of dehydration, the moisture content of the wood ear mushrooms significantly decreased at all temperatures; the moisture content at 70, 80, and 90°C (64.26 ± 4.38, 66.91 ± 3.02, and 54.65 ± 5.31%, respectively) were not significantly different. After 1 h of dehydration, the moisture content at 90°C (20.96 ± 5.84%) was significantly lower than that at the other two temperatures. After 2 h of dehydration, the moisture content of the wood ear mushrooms equilibrated across temperatures; there was no significant difference between the moisture contents at 70, 80, or 90°C (12.40 ± 1.92, 14.38 ± 3.47, and 11.68 ± 3.50%, respectively). Dehydration for longer time periods also did not result in a significant decrease in the moisture content of the wood ear mushrooms.

### 3.2. Pathogen inactivation on enoki mushrooms during dehydration

**Figure 2:**
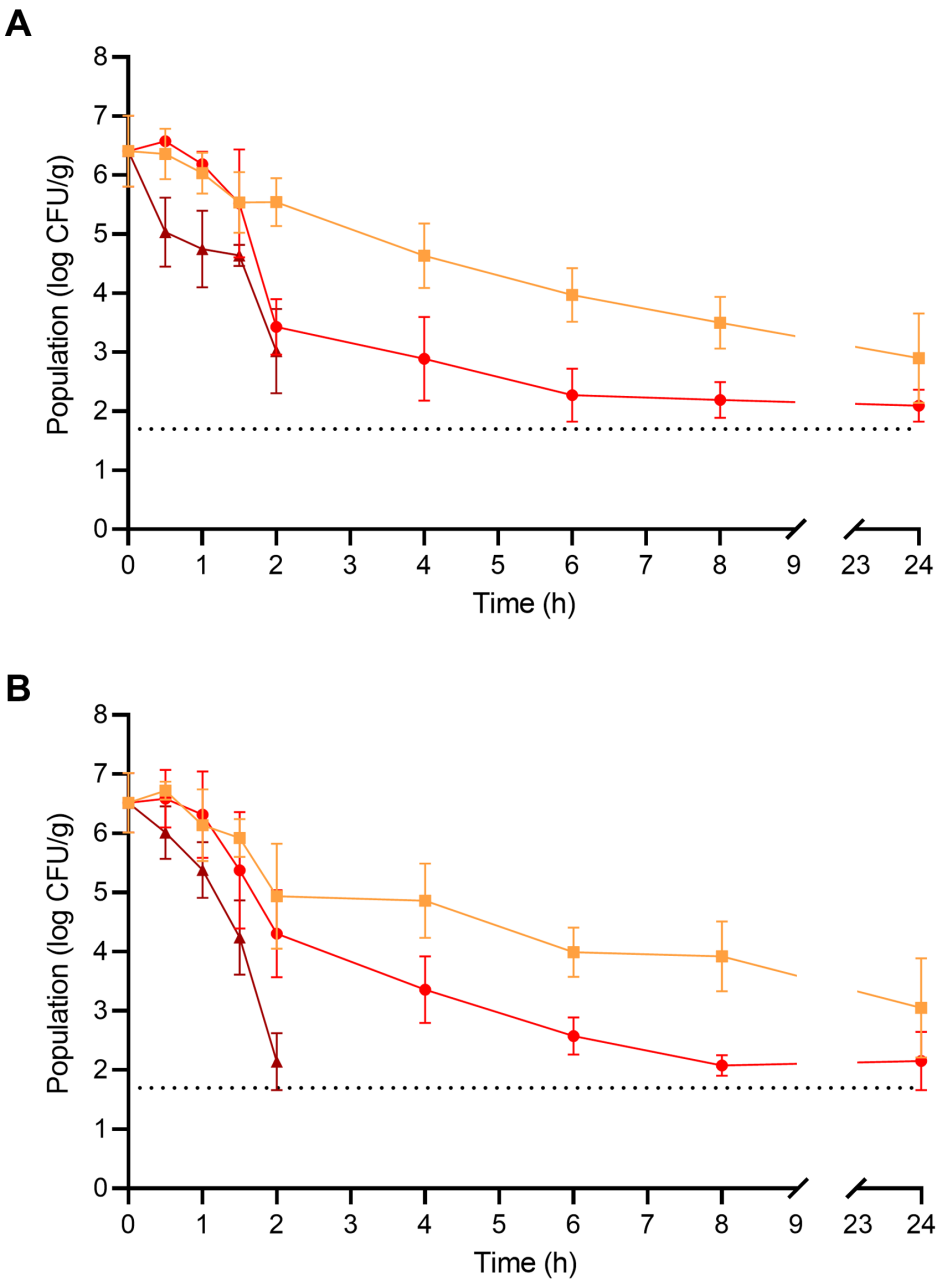
The populations of **(A)** *L. monocytogenes* and **(B)** *S. enterica* on enoki mushrooms during dehydration at 70 (orange square), 80 (red circle), and 90°C (brown triangle) for 24 h. The dotted line indicates the lower level of enumeration (1.7 log CFU/g). Data are mean values ± standard deviation (*n* = 9).

The populations of *L. monocytogenes* and *S. enterica* on the fresh enoki mushrooms prior to dehydration were 6.40 ± 0.60 and 6.51 ± 0.50 log CFU/g, respectively (Figure 2). For *L. monocytogenes*, significant population reductions were observed on enoki mushrooms dehydrated at 90°C after only 0.5 h (where the population was reduced by 1.36 log CFU/g), whereas at 70 and 80°C, significant population reductions were observed after 4 and 2 h, respectively (reductions of 1.77 and 2.97 log CFU/g, respectively). After 2 and 4 h of dehydration at 90°C, the population of *L. monocytogenes* was reduced by 3.38 and ≥ 4.70 log CFU/g, respectively. A tailing effect was observed during dehydration of enoki mushrooms at both 70 and 80°C, with remaining populations of *L. monocytogenes* of 3.50 ± 0.43 and 2.19 ± 0.30 log CFU/g, respectively (corresponding to reductions of 2.90 and 4.21 log CFU/g, respectively).

For *S. enterica*, significant population reductions were observed on enoki mushrooms dehydrated at 80 and 90°C after only 2 and 1.5 h (population reductions of 2.21 and 2.26 log CFU/g, respectively), whereas at 70°C, a significant population reduction was observed after 6 h (reduction of 2.52 log CFU/g). After 2 and 4 h of dehydration at 90°C, the population of *S. enterica* was reduced by 4.37 and ≥ 4.70 log CFU/g, respectively. As observed with *L. monocytogenes*, a similar tailing effect occurred with *S. enterica* during dehydration of enoki mushrooms at both 70 and 80°C, with remaining populations of 3.92 ± 0.59 and 2.08 ± 0.17 log CFU/g, respectively (corresponding to reductions of 2.59 and 4.43 log CFU/g, respectively).

The Weibull model was first utilized to describe the inactivation of *L. monocytogenes* and *S. enterica* on enoki mushrooms during dehydration at all temperatures examined (Table 1). The parameter δ, or the time for a 1 log CFU/g reduction in population ranged from 0.39–1.33 h, depending on the pathogen and temperature. The shape parameter *ρ* (0.39–0.71) indicated that the survival of *L. monocytogenes* and *S. enterica* on enoki mushrooms followed an upward concavity at all temperatures, with the exception of *S. enterica* at 90°C (*ρ* = 1.95), indicating a convex survival curve. According to the Weibull model, a 4 log CFU/g reduction of *L. monocytogenes* would occur after 24.00, 14.16, and 3.16 h on enoki mushrooms during dehydration at 70, 80, and 90°C, respectively. While a 4 log CFU/g reduction of *S. enterica* was not determined to occur at 70°C, reductions would occur at 80 and 90°C after 9.12 and 1.96 h, respectively.

**Table 1:** Kinetic parameters of the Weibull model to describe the inactivation of *L. monocytogenes* and *S. enterica* on enoki and wood ear mushrooms during dehydration at 70, 80, or 90°C.

Mushroom	Pathogen	Temperature (°C)	δ ± SE (h)	*ρ* ± SE	*N*_0_ ± SE (log CFU/g)	*r* ^2^	MSE	4D reduction (h)
Enoki	*L. monocytogenes*	70	1.28 ± 0.34	0.47 ± 0.04	6.51 ± 0.10	0.79	0.3898	24.00
		80	0.39 ± 0.21	0.39 ± 0.06	6.41 ± 0.15	0.76	0.9028	14.16
		90	0.44 ± 0.10	0.71 ± 0.10	6.40 ± 0.10	0.77	0.4065	3.16
	*S. enterica*	70	1.33 ± 0.36	0.47 ± 0.05	6.61 ± 0.10	0.75	0.4381	ND
		80	0.52 ± 0.16	0.49 ± 0.05	6.69 ± 0.13	0.75	0.7721	9.12
		90	0.96 ± 0.07	1.95 ± 0.23	6.49 ± 0.08	0.82	0.2643	1.96
Wood ear	*L. monocytogenes*	70	0.96 ± 0.16	0.70 ± 0.06	6.34 ± 0.09	0.88	0.2290	6.96
		80	0.62 ± 0.10	0.85 ± 0.09	6.30 ± 0.11	0.83	0.3231	3.16
		90	0.72 ± 0.07	1.48 ± 0.15	6.29 ± 0.09	0.90	0.2518	1.86
	*S. enterica*	70	0.88 ± 0.10	1.00 ± 0.08	6.42 ± 0.09	0.90	0.2469	3.52
		80	0.70 ± 0.11	0.87 ± 0.08	6.45 ± 0.11	0.86	0.3135	3.44
		90	0.80 ± 0.08	1.64 ± 0.19	6.40 ± 0.10	0.86	0.3059	1.88

SE, standard error; δ, time (h) for the first decimal reduction in population; *ρ*, shape parameter; *N*_0_, initial population (log CFU/g); *r*^2^, coefficient of determination; SME, mean square error; 4D reduction (h), time for a 4 log CFU/g reduction; ND, none determined.

Secondly, the log-linear model, with either tail or shoulder, was also used to describe the inactivation of *L. monocytogenes* and *S. enterica* on enoki mushrooms during dehydration (Table 2). Due to the population tailing effects observed by both pathogens during dehydration at 70 and 80°C, the log-linear with tail model was utilized. Residual populations of both pathogens remained on enoki mushrooms after 24 of dehydration at 70 and 80°C (2.18–2.97 log CFU/g for *L. monocytogenes* and 2.25–3.25 log CFU/g for *S. enterica*). While a 4 log CFU/g decrease in population was not predicted to occur for either pathogen at 70°C, 4 log CFU/g decreases were determined to occur at 80°C after 5.04 and 5.28 h for *L. monocytogenes* and *S. enterica*, respectively. No tailing was observed for *S. enterica* on enoki mushrooms during dehydration at 90°C, and hence the log-linear with shoulder model was used; the maximum inactivation rate was 6.35 ± 0.71 log CFU/g/h. For *L. monocytogenes*, neither of the log-linear models adequately described the inactivation.

**Table 2:** Kinetic parameters of the log-linear with tail model (for 70 and 80°C) and the log-linear with shoulder model (for 90°C) to describe the inactivation of *L. monocytogenes* and *S. enterica* on enoki and wood ear mushrooms during dehydration.

Mushroom	Pathogen	Temperature (°C)	Sl ± SE (h)	*k*_max_ ± SE (log CFU/g/h)	*N*_0_ ± SE (log CFU/g)	*N*_res_ ± SE (log CFU/g)	*r* ^2^	MSE	4D reduction (h)
Enoki	*L. monocytogenes*	70	NA	0.95 ± 0.06	6.39 ± 0.07	2.97 ± 0.17	0.84	0.2897	ND
		80	NA	2.06 ± 0.16	6.50 ± 0.10	2.18 ± 0.16	0.89	0.4115	5.04
		90	NA	NA	NA	NA	NA	NA	NA
	*S. enterica*	70	NA	0.97 ± 0.08	6.50 ± 0.08	3.25 ± 0.19	0.79	0.3771	ND
		80	NA	1.89 ± 0.13	6.63 ± 0.08	2.25 ± 0.17	0.87	0.4017	5.28
		90	0.61 ± 0.10	6.35 ± 0.71	6.48 ± 0.08	NA	0.81	0.2840	ND
Wood ear	*L. monocytogenes*	70	NA	2.33 ± 0.17	6.38 ± 0.08	3.08 ± 0.11	0.89	0.1987	ND
		80	NA	3.44 ± 0.22	6.32 ± 0.09	1.89 ± 0.39	0.84	0.2922	2.84
		90	0.44 ± 0.09	6.53 ± 0.49	6.25 ± 0.09	NA	0.90	0.2408	1.86
	*S. enterica*	70	NA	2.85 ± 0.19	6.48 ± 0.09	1.75 ± 0.31	0.91	0.2408	3.32
		80	NA	3.35 ± 0.21	6.52 ± 0.09	2.16 ± 0.22	0.87	0.2616	2.96
		90	0.55 ± 0.10	6.88 ± 0.65	6.35 ± 0.10	NA	0.87	0.2984	1.90

Sl, shoulder length (h); SE, standard error; k_max_, maximum inactivation rate (log CFU/g/h); *N*_0_, initial population (log CFU/g); N_res_, remaining or residual population (log CFU/g); *r*^2^, coefficient of determination; SME, mean square error; 4D reduction (h), time for a 4 log CFU/g reduction; NA, not application; ND, none determined.

### 3.3. Pathogen inactivation on wood ear mushrooms during dehydration

**Figure 3:**
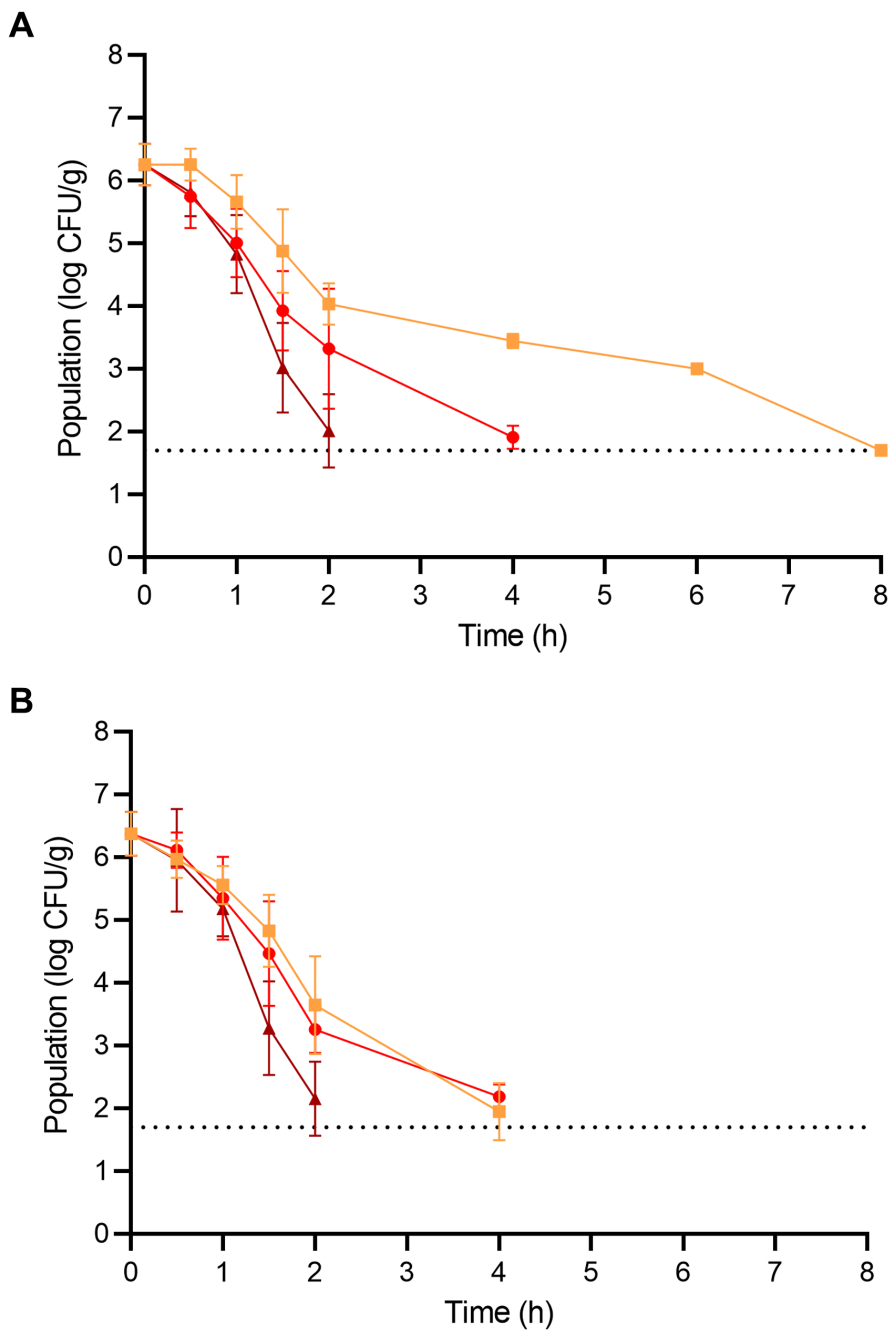
The populations of **(A)** *L. monocytogenes* and **(B)** *S. enterica* on wood ear mushrooms during dehydration at 70 (orange square), 80 (red circle), and 90°C (brown triangle) for 8 h. The dotted line indicates the lower level of enumeration (1.7 log CFU/g). Data are mean values ± standard deviation (*n* = 9).

The populations of *L. monocytogenes* and *S. enterica* on the fresh wood ear mushrooms prior to dehydration were 6.26 ± 0.33 and 6.38 ± 0.35 log CFU/g, respectively (Figure 3). For *L. monocytogenes*, significant population reductions were observed on wood ear mushrooms after 1.5 h at 70°C (reduction of 1.37 log CFU/g) and after only 1 h at both 80 and 90°C (reductions of 1.25 and 1.43 log CFU/g, respectively). After 2 and 4 h of dehydration at 90°C, the population of *L. monocytogenes* was reduced by 4.25 and ≥ 4.56 log CFU/g, respectively. After 4 and 6 h of dehydration at 80°C, the population of *L. monocytogenes* was reduced by 4.35 and ≥ 4.56 log CFU/g, respectively. At 70°C, a 3.26 log CFU/g reduction in population was observed after 6 h, followed by a reduction of ≥4.56 log CFU/g after 8 h.

For *S. enterica*, significant population reductions were observed on wood ear mushrooms after 1 h of dehydration at all temperatures; population reductions after 1 h were 0.82, 1.03, and 1.20 log CFU/g at 70, 80, and 90°C, respectively. After 2 and 4 h of dehydration at 90°C, the population of *S. enterica* was reduced by 4.22 and ≥ 4.68 log CFU/g, respectively. After 4 h of dehydration at 70 and 80°C, the population of *S. enterica* was reduced by 4.43 and 4.19 log CFU/g, respectively. A reduction of ≥4.68 log CFU/g was observed on wood ear mushrooms after dehydration at both 70 and 80°C after 6 h.

As with enoki mushrooms, the Weibull model was first utilized to describe the inactivation of *L. monocytogenes* and *S. enterica* on wood ear mushrooms during dehydration (Table 1). The time for a 1 log CFU/g reduction in population ranged from 0.62–0.96 h, depending on the pathogen and temperature. The shape parameter *ρ* indicated that the survival of *L. monocytogenes* on wood ear mushrooms during dehydration at both 70 and 80°C, along with *S. enterica* at 80°C, followed an upward concavity. *S. enterica* survival at 70°C was predicted to be log-linear (*ρ* = 1.00). The survival curves for both pathogens on wood ear mushrooms during dehydration at 90°C were convex. According to the Weibull model, a 4 log CFU/g reduction of *L. monocytogenes* would occur on wood ear mushrooms after 6.96, 3.16, and 1.86 h during dehydration at 70, 80, and 90°C, respectively. Similarly, for *S. enterica*, 4 log CFU/g reductions would occur after 3.52, 3.44, and 1.88 h during dehydration at 70, 80, and 90°C, respectively.

Subsequently, the log-linear model, with either tail or shoulder, was also used to describe the inactivation of *L. monocytogenes* and *S. enterica* on wood ear mushrooms during dehydration (Table 2). Although minimal or no population tailing was observed to occur on wood ear mushrooms at 70 or 80°C, the log-linear with tail model predicted residual populations of 1.89–3.08 log CFU/g for *L. monocytogenes* and 1.75–2.16 log CFU/g for *S. enterica*. While a 4 log CFU/g decrease in population was not predicted to occur for *L. monocytogenes* at 70°C, a 4 log CFU/g decrease was determined to occur at 80°C after 2.84 h. For *S. enterica*, 4 log decreases were determined to occur after 3.32 and 2.96 h at 70 and 80°C. For 90°C, the log-linear with shoulder model was used to predict the survival of both pathogens on wood ear mushrooms during dehydration. Decreases of 4 log CFU/g were predicted to occur at 90°C after 1.86 and 1.90 h for *L. monocytogenes* and *S. enterica*, respectively.

## 4. Discussion

This study evaluated the survival and inactivation kinetics of *L. monocytogenes* and *S. enterica* on two types of specialty mushrooms during dehydration at. Dehydration of fresh produce, including mushrooms, is a common method used by both the food industry and consumers to extend the shelf life of produce while also preserving quality. Since dehydration is generally conducted at low temperatures, it is not used as a preventive control to inactivate bacterial foodborne pathogens. Therefore, this study evaluated the effect of dehydration at the higher temperatures of 70, 80, and 90°C on pathogen inactivation on enoki and wood ear mushrooms. It is noted that the results reported in this study are specific to the drying conditions used, the sample size and layout in the dehydrators, and the initial moisture contents of the mushrooms.

Pathogen inactivation during dehydration was dependent on the mushroom type as well as the dehydration time and temperature combination. For enoki mushrooms, this study observed similar pathogen reductions as when portobello mushrooms were dehydrated at 55°C for 8 h (Kragh et al., 2022). Log reductions of 2.91 and 2.60 log CFU/g for *L. monocytogenes* and *S. enterica*, respectively, on enoki mushrooms were obtained in this study during dehydration at 70°C for 8 h (with no significant further reduction after 24 h). While 80°C resulted in a greater reduction of both pathogens on enoki mushrooms, residual populations were still observed after 24 h (based on the initial inoculation level of 6.5 log CFU/g). The tailing effect observed at both 70 and 80°C was most likely attributed to the increased thermal resistance of the pathogens during dehydration at those temperatures. This is not surprising as the lower moisture content of the food matrix would result in increased thermal resistance of both pathogens.

Dehydration at the enoki and wood ear mushrooms at 90°C resulted in the highest pathogen inactivation, >4 log CFU/g, after only 2–4 h. The moisture contents of both mushrooms were also significantly lower after 0.5, 1, and 1.5 h of dehydration at 90°C compared to the other two temperatures. Interestingly, pathogen reductions were significantly higher on wood ear mushrooms at 70°C: reductions of >4 log CFU/g occurred for both pathogens after only 4–8 h, despite similar moisture contents of these mushrooms compared to enoki. Dehydration at the higher temperatures of 80 and 90°C resulted in reductions >4 log CFU/g in 4 h or less. While a tailing effect was observed for both pathogens on enoki mushrooms at both 70 and 80°C, the same tailing effect was not observed on wood ear mushrooms. The moisture contents for both enoki and wood ear mushrooms equilibrated after 2–4 h regardless of the dehydration temperature. The greater pathogen reduction on wood ear mushrooms during dehydration may be attributed to their antimicrobial compounds, including melanin and polyphenols (Cai et al., 2015; Liu et al., 2021), which have been shown to inhibit both the proliferation and biofilm-forming abilities of various pathogens. In a previous study, it was determined that a 1-h ambient drying of *L. monocytogenes* and *S. enterica* on dehydrated wood ear mushrooms resulted in reductions of 2.49 and 2.26 log CFU/g, respectively, whereas no significant decrease was observed on enoki mushrooms under the same conditions (Fay et al., 2023). Antimicrobial compounds have been known to influence pathogen *D* values; the antimicrobial properties of chili powder, for example, significantly increased the *D* values of *S. enterica* during 70°C heat treatment at a_w_ 0.3, compared to those of ground cinnamon or black pepper powder (Xie et al., 2022).

This study determined that for enoki mushrooms, dehydration temperatures below 90°C did not achieve >4 log CFU/g reduction of either *L. monocytogenes* or *S. enterica*, even after 24 h. In response to the listeriosis outbreak in the U.S. in 2020 associated with enoki mushrooms, traceback and laboratory investigations determined that the implicated mushrooms contained levels of *L. monocytogenes* as high as 5.90 log CFU/g (Pereira et al., 2023). *L. monocytogenes* has also been identified in mushrooms in retail markets in Spain (Venturini et al., 2011) and China (Chen et al., 2016) and in mushroom production or processing facilities in the U.S. (Viswanath et al., 2013; Murugesan et al., 2015). In addition, high levels of both background microbiota and *Enterobacteriaceae* have also been found on other types of mushrooms at retail markets (Venturini et al., 2011). Wood ear mushrooms (*Auricularia auricula-judae*) had the highest microbial load (9.4 log CFU/g) out of 22 tested mushroom varieties from retail markets in Spain; wood ear also had the highest *Enterobacteriaceae* population (6.5 log CFU/g) (Venturini et al., 2011). In the same study, the total microbial load and *Enterobacteriaceae* populations on enoki mushrooms (*Flammulina velutipes*) were 7.1 and 5.9 log CFU/g, respectively. Taken together with the published literature, the results of this study and the specific conditions examined suggest that the use of dehydration alone at temperatures below 90°C would not be an effective preventive control to ensure the safety of all mushrooms.

It is noted that the use of higher drying temperatures have been shown to negatively affect the organoleptic properties or the rehydration ratio for some mushrooms; for example, button mushrooms dehydrated at 50°C, compared to 70°C, had better rehydration characteristics and were lighter in color (Nour et al., 2011). However, dehydration at 70 and 80°C, compared to lower temperatures, was able to retain the sensory profiles of oyster and shiitake mushrooms, respectively (Politowicz et al., 2018a,b). The influence of the higher dehydration temperatures on the quality characteristics of mushrooms may therefore vary between mushroom type. While the quality characteristics of the enoki and wood ear mushrooms were not formally evaluated in this study, dehydration at all tested temperatures resulted in a visually observed browning of the enoki mushrooms. No noticeable color change was observed for the wood ear mushrooms. Mushroom type-specific evaluations would need to be conducted to find a balance which preserves the mushroom quality while also maintaining safety.

In conclusion, this study aimed to understand the inactivation kinetics of both *L. monocytogenes* and *S. enterica* on enoki and wood ear mushrooms during dehydration. Of the three temperatures evaluated, 90°C was most effective at pathogen reduction on both mushrooms, while temperatures of 70 and 80°C were not as effective for enoki mushrooms. Due to the striking difference in the inactivation of both pathogens between the two types of specialty mushrooms, this study highlights the need for individual mushroom type assessments. Results from this study suggest that dehydration at the examined temperatures would not serve as a preventive control for specialty mushrooms.

## Data availability statement

The original contributions presented in the study are included in the article/supplementary material, further inquiries can be directed to the corresponding author.

## Author contributions

JS: Conceptualization, Data curation, Formal analysis, Investigation, Methodology, Supervision, Writing – original draft, Writing – review & editing. MF: Data curation, Formal analysis, Investigation, Methodology, Writing – original draft, Writing – review & editing. BK: Data curation, Investigation, Methodology, Writing – review & editing. NC: Data curation, Investigation, Methodology, Writing – review & editing. GP: Data curation, Investigation, Methodology, Writing – review & editing. DI: Data curation, Investigation, Writing – review & editing.
